# A mixed-methods exploration of stakeholder experiences and perspectives on integrating polygenic breast cancer risk scores into Swedish clinical practice

**DOI:** 10.1186/s12913-026-15116-7

**Published:** 2026-07-11

**Authors:** Sergio Flores, Kellie Harkin, Filipa Sampaio, Evangelos Digkas, Andreas Karakatsanis, Helena Lundgren, Madli Tamm, Peeter Padrik, Krista Kruuv-Käo, Siim Sõber, Magda Rosenmöller, Neeme Tõnisson, Inna Feldman

**Affiliations:** 1 Uppsala Health Economics, Department of Public Health and Caring Sciences, Uppsala University, Uppsala, Sweden; 2 Region Uppsala, Uppsala, Sweden; 3 Department of Women’s and Children’s Health, Uppsala University, Uppsala, Sweden; 4 Department of Learning, Informatics, Management and Ethics, Karolinska Institute, Stockholm, Sweden; 5 Department of Immunology, Genetics, and Pathology, Uppsala University, Uppsala, Sweden; 6 Department of Oncology, Uppsala University Hospital, Uppsala, Sweden; 7 Department for Surgical Sciences, Faculty of Medicine, Uppsala University, Uppsala, Sweden; 8 Section for Breast Surgery, Department of Surgery, Akademiska University Hospital, Uppsala, Sweden; 9 Institute of Genomics, University of Tartu, Tartu, Estonia; 10 Genetics and Personalized Medicine Clinic, Tartu University Hospital, Tartu, Estonia; 11 OÜ Antegenes, Tartu, Estonia; 12 IESE Business School, University of Navarra, Barcelona, Spain; 13 Clinic of Haematology and Oncology, Tartu University Hospital, Tartu, Estonia

**Keywords:** Polygenic risk score, Breast cancer, Clinical practice, Stakeholders, Mixed-methods

## Abstract

**Objective:**

To explore women’s experiences following polygenic risk score (PRS) testing for breast cancer risk and to assess the knowledge, attitudes, practices (KAP), and implementation perspectives of medical professionals and healthcare decision-makers in Sweden.

**Methods:**

Convergent mixed-methods study combining quantitative survey data with qualitative thematic analysis of survey open-ended responses and focus group discussion (FGD) notes. Setting: Swedish healthcare context. Participants: 400 women receiving PRS results for a first Participants Feedback Survey; 289 women for the second Participants Feedback Survey, and 6 medical professionals/decision-makers for a KAP survey and FGD. Women completed two sequential online feedback surveys post-PRS result disclosure. Professionals completed a KAP survey and participated in an FGD. Quantitative survey data were analysed using descriptive statistics. Qualitative data from open-ended survey questions and FGD notes were analysed using thematic analysis.

**Results:**

Participants valued participation in PRS testing and receiving PRS-test results, with 87% finding results interesting and 82% finding them valuable. Although most participants found the explanations understandable (approximately 75% in Survey 1 and 82% in Survey 2), qualitative comments indicated that some had difficulty interpreting probabilistic risk information, including participants who self-identified as highly educated or medically trained. Negative emotional impact was generally minimal (85% felt calm), though some women with high PRS risk experienced anxiety. Major unmet needs included clearer explanations, actionable guidance, and better access to follow-up support from healthcare. Professionals were cautiously positive: in the KAP survey, 5 of 6 were familiar with the concept of PRS, but confidence in the health system’s readiness to integrate it was limited. The main barriers raised were the absence of clinical guidelines and the need for evidence of clinical utility.

**Conclusions:**

Breast cancer PRS testing holds potential for enhancing risk assessment in Sweden. Key challenges for clinical integration include clinician readiness, the development of clear guidelines, and improved participant comprehension and communication. PRS tests should be accompanied with clinical decision support both for patients as well for medical professionals. Addressing these will require person-centered communication tools, robust evidence of clinical utility, well-defined clinical pathways, investments in provider education, and equitable implementation strategies within the Swedish healthcare system.

**Clinical trial number:**

Not applicable.

**Supplementary Information:**

The online version contains supplementary material available at 10.1186/s12913-026-15116-7.

## Introduction

Breast cancer (BC) remains a significant cause of morbidity and mortality for women worldwide, including in Sweden [1–3]. Although high-penetrant variants such as BRCA1/2 are important, they account for less than a quarter of the familial relative risk [4, 5], leaving much heritability unexplained This remaining risk is largely due to the combined effects of many common, low-penetrance SNPs identified through genome-wide association studies (GWAS) [6, 7]. Polygenic risk scores integrate these variants to estimate inherited susceptibility on a continuous scale, providing a probabilistic risk assessment rather than a single-gene, deterministic result [5, 7–9].

Despite this potential, widespread clinical implementation of PRS testing faces significant challenges. A critical limitation is the pronounced ancestry bias; PRSs have been developed predominantly in European populations, resulting in reduced performance and poor calibration in individuals of non-European ancestry, which raises significant equity concerns [5, 10, 11]. Furthermore, demonstrating definitive clinical utility—evidence that PRS use improves patient outcomes or healthcare efficiency compared to standard care—requires ongoing large-scale trials. A recently published national randomized control trial (The WISDOM study) demonstrated feasibility for scaled implementation of PRS screening for BC, as well as moderate changes to individual screening recommendations, and minimal projected downstream burden on the healthcare system [12]. However, while the clinical potential of PRS has been established, its broader adoption requires an understanding of the key stakeholders’ perspectives/viewpoints.

Medical professionals, particularly outside the medical specialties of genetics or medical oncology, often report limited familiarity with PRS and require further education [13, 14]. While recognizing PRS’s testing potential for identifying high-risk individuals, clinicians express significant hesitation regarding its use to de-intensify screening for those at lower risk, often citing the lack of clear clinical guidelines [9, 13]. Patients and the public show considerable interest but frequently struggle with interpreting probabilistic risk information and report concerns about access to follow-up care, cost, potential genetic discrimination, data privacy, and equity [15–17]. Policymakers are exploring PRS testing for optimising screening programs, but await robust evidence on clinical utility, cost-effectiveness, and strategies for equitable implementation within complex healthcare systems [12, 18, 19]. The ethical, legal, and social implications (ELSI) surrounding communication, equity, discrimination, and data governance require careful navigation [5, 17, 20].

Given the potential of PRS testing to refine BC risk assessment and the identified barriers to its implementation, understanding specific contextual factors is essential. To explore the use of PRS in BC screening, the multicentre “Be RIGHT with breast cancer risk management” (BRIGHT) study was initiated and spanned three countries—Portugal, Estonia, and Sweden—concluding in December 2024. The BRIGHT study was a research and innovation initiative aimed at developing and evaluating the feasibility of a new genetics-based BC screening service utilizing a PRS test developed by Antegenes [21–23]. The results of the clinical BRIGHT study in Estonia demonstrated the feasibility and acceptability of a personalised genetic risk-based BC screening model using PRS testing [23]. The results of Swedish and Portuguese clinical studies will be published separately. This Swedish study aimed to explore the experiences and acceptance of the BRIGHT study participants following receiving the BC PRS results, and to assess the knowledge, attitudes, practices, and implementation perspectives of relevant Swedish medical professionals and healthcare decision-makers.

## Methods

### Study design

We employed a convergent (parallel) mixed-methods design [24], in which quantitative and qualitative strands were collected concurrently, analysed separately, and merged during interpretation. Quantitative survey data were collected and analysed alongside qualitative data. This included data from an open-ended survey delivered to the recruited BRIGHT study participants after receiving their PRS estimates for breast cancer risk, as well as a focus group discussion (FGD) conducted with healthcare professionals and decision-makers to explore implementation perspectives and barriers for PRS integration into clinical practice. This approach allowed for a comprehensive exploration of participants’ experiences post-PRS testing and professional perspectives regarding PRS implementation within the Swedish context.

### Recruitment and data collection

#### BRIGHT-study participants

In total, 793 women residing in Sweden participated in the Swedish BRIGHT study. The study cohort consisted of healthy women aged 30–49, a part of them aged 30–39 was not yet attending regular BC screening. Participants were recruited from March 2023 to April 2024 via online advertisements on social media platforms, local media, and websites. Following expression of interest, women registered and provided informed consent through a secure REDCap portal and completed a recruitment questionnaire capturing personal data, family history of cancer, reproductive factors, and anthropometric measures; DNA was collected using a mailed, self-administered buccal-swab kit (OU Antegenes) returned by prepaid envelope. Detailed descriptions of the recruitment procedures, sampling strategy, and cohort characteristics are beyond the scope of this paper and will be presented in a separate clinical manuscript.

#### PRS result reports and recommendations

PRS-test results were delivered via email as comprehensive personalised risk reports generated based on the AnteBC test (OÜ Antegenes, Estonia; see Appendix, Figure A1). These reports included the participant’s PRS-based estimation of risk for BC expressed both as standard deviation units (e.g., 0.12 SD) and percentile ranking (e.g., 55th percentile), absolute 10-year risk estimates (e.g., 1.19%), relative risk compared to a population average (e.g., 1.05 times higher), and visual representations including population comparison charts and genetic variant breakdowns showing both risk-increasing and risk-decreasing variants. PRS results (expressed as relative risk, RR) were summarized across the study population and stratified into three risk categories based on their relative risk compared to the general population: low/average (RR < 1.5), slightly elevated (1.5 ≤ RR < 2.7), and elevated (RR > 2.7). Participants in the “slightly elevated” and “elevated” risk groups were offered per the study protocol a consultation with a specialist at the Breast Unit at Uppsala University Hospital. In total, 71 participants booked a clinical consultation: 2 (0.3%) in the low-risk group, 61 (47.7%) in the slightly elevated-risk group, and 8 (72.7%) in the high-risk group. Consultations were conducted by a breast surgeon or a clinical geneticist. While email was the primary communication channel, phone consultations were also available.

#### Consultation with specialists

During the consultation, a pedigree was created that included all relevant malignant diagnoses in the family. The lifetime risk for breast cancer was estimated using the CanRisk tool [25], and a referral for baseline mammography was provided if the participant had not undergone breast cancer screening in the year prior to the consultation. This mammography referral applied only to women attending a consultation (i.e., the slightly elevated and elevated risk groups); women in the low/average risk group received a recommendation to begin biennial screening from age 40. Breast density, where available, was recorded at the clinical examination, but mammography was not performed specifically to ascertain density for the CanRisk assessment. Afterward, the pedigree data and the CanRisk results were reviewed by a medical geneticist, following the national Swedish guidelines. If the clinical criteria for familial cancer were met, the patient was recommended for referral to the medical genetics’ unit for genetic counselling. Finally, all participants received a letter summarizing the results and all given recommendations, see Appendix, Figure A1, A2, A2a, A2b, A2c).

#### Feedback surveys

Participants’ feedbacks were collected via two sequential online surveys administered at distinct time points. The surveys were developed for this study and presented in the Appendix (Participant’s Feedback Survey 1 and Participant’s Feedback Survey 2). The first survey (Feedback Survey 1), administered 4–6 weeks after PRS result disclosure, was completed by 400 women. The second survey (Feedback Survey 2), administered 9 months after receiving the PRS report, was completed by 289 women. Within the Survey 2 cohort, approximately 80% (193 of 241 who answered the relevant item) indicated they had also completed Survey 1. All survey instruments were developed specifically for this study and administered via REDCap; they did not incorporate previously validated scales. Items were informed by the existing literature and the clinical and research expertise of the project team, and the questionnaires were not formally piloted before use. Participants were asked to provide feedback on their overall experience of receiving and understanding their PRS results (including emotional response, comprehension of the report, the adequacy of the information and communication provided, decision satisfaction, and the need for follow-up), rather than on any single step of the process.

#### Professionals/decision-makers

This group comprised medical professionals (oncologists, geneticists, radiologists) and health system stakeholders (biomedical engineers, managers, decision-makers) with roles related to BC care, screening, medical genetics, and health technology assessment in Sweden. Six professionals completed an online Knowledge, Attitudes, and Practices (KAP) survey, recruited via purposive sampling. The same six professionals (Medical Geneticist, Biomedical Engineer, Oncologist, Breast Radiologist/Researcher, Mammography Unit Manager, Mammogram Department Secretary) participated in a single FGD, invited based on their specific expertise relevant to PRS implementation into clinical practice. Before completing the KAP survey and the FGD, the professionals attended a half-day project workshop (13 June 2023) that included a presentation introducing the BRIGHT study and PRS, including cost-effectiveness, a progress update on the Uppsala clinical study, and a question-and-answer session. No targeted educational or training intervention on PRS was delivered.

### Data collection instruments

#### Participants’ feedback surveys

Two online surveys deployed via REDCap V 15.0.33 captured participant experiences. Survey 1 (short-term follow-up) aimed to assess emotional responses, acceptability, and feasibility of PRS-based BC screening. The questionnaire included 19 items addressing the clarity and sources of information, digital tools usage, emotional responses to test results, informational needs and comprehension of the polygenic risk, satisfaction with participation, and intentions for further suggested medical action. Most questions were multiple-choice, utilizing a five-point Likert scale, with optional free-text responses for specific topics (see Appendix, Participant’s Feedback Survey 1). Survey 2 (long-term follow-up) included questions about psychological responses, decision regret, coping, perceived control, and with whom participants shared the results. It also included extensive open-ended fields for detailed feedback on understanding, communication needs, and suggestions for improvement (see Appendix, Participant’s Feedback Survey 2).

#### Professional KAP survey

An online survey deployed via REDCap V 15.0.33 assessed professionals’ PRS familiarity, definitions used, information sources, perceptions regarding accuracy, reliability, and integration importance, views on benefits and limitations, factors influencing clinical inclusion, perceived challenges, implementation readiness, communication strategies, and barriers. The instrument included Likert-type scales, multiple-choice questions, and open-ended text fields (see Appendix KAP-survey).

#### Focus group discussion (FGD)

A single, approximately 1.5-hour, mediated FGD was conducted on June 13th, 2023, with the six professionals immediately following their completion of the KAP survey. The discussion guide covered: ideal PRS implementation scenarios, reasonable timelines, evidence requirements for adoption, perceived external and systemic barriers, and key ethical or legal considerations. Detailed notes were compiled by three designated observers, capturing main discussion points and attributed statements where feasible.

### Data analysis

We integrated findings from the two temporally distinct participant feedback surveys (Survey 1: *n* = 400, initial post-result; Survey 2: *n* = 289, 9-month follow-up), as well as from the professional KAP survey (*n* = 6), and the professional FGD (*n* = 6, same participants as the KAP survey).

Quantitative data derived from closed-ended surveys questions (Likert scales, categorical responses) were analysed using descriptive statistics. Descriptive statistics were used because the study was exploratory, the samples were small and of unequal size (Survey 1 *n* = 400; Survey 2 *n* = 289; professionals *n* = 6), and the two participant surveys could not be linked at the individual level, precluding paired within-person comparison; inferential testing of change over time and of associations between variables was therefore not appropriate at this scale. Frequencies and percentages were calculated for categorical variables, while means and standard deviations (SD) were computed for scale variables. Qualitative data consisted of all open-ended responses from the three surveys (KAP, Survey 1, Survey 2) and the detailed FGD notes. These data were analysed using thematic analysis. Two researchers (SF and KH) independently read through the textual data to gain familiarity, developed initial codes representing meaningful segments of text, and then collaboratively grouped these codes into broader themes. Themes were reviewed, discussed, and refined iteratively to ensure they accurately reflected the dataset. The candidate themes were subsequently reviewed and discussed with two further members of the research team (IF and FS) before being finalised. Connections and divergences in themes across participant groups (professionals, participants) and data sources (surveys, FGD) were examined. Illustrative participant quotes, translated from Swedish where necessary, were selected to exemplify key themes.

Integration of the quantitative and qualitative strands followed the convergent mixed-methods approach described by Fetters and colleagues [26], using a side-by-side comparison (joint display) in which quantitative survey findings and qualitative themes were considered alongside across the two stakeholder groups (participants and professionals) and data sources (surveys and FGD) to identify convergence, divergence, and expansion. Because the participant feedback surveys could not be reliably linked to one another at the individual level, integration and comparison were undertaken at the group and interpretive level rather than the individual level. The Synthesis Across Perspectives section presents this integration.

Reporting follows the Good Reporting of a Mixed Methods Study (GRAMMS) guideline [27] for the overall study and the Consolidated Criteria for Reporting Qualitative Research (COREQ) [28] for the qualitative component; completed checklists are provided as related files. Perceived validity was addressed in line with established qualitative criteria [29]: credibility, through independent dual coding by two researchers (SF and KH) with iterative, consensus-based theme refinement and the use of illustrative participant quotations; dependability, through a documented and systematic coding process; and confirmability, by grounding themes in the raw textual data and examining convergence and divergence across data sources. The FGD was captured through structured observer notes rather than verbatim transcription, and member checking and a formal audit trail were not undertaken; these are acknowledged as limitations.

### Ethical considerations

Participation in all study components was voluntary. Consent was implied by survey completion and obtained verbally prior to the FGD. To ensure anonymity, all directly identifiable information collected in the participant surveys (names, email addresses, phone numbers, specific participant identifiers) was removed prior to analysis and is excluded from this report. This study was performed in the frame of the BRIGHT study, which had ethical approval from the Swedish Ethical Review Authority (Dnr. 2022-03074-01).

## Results

### Participant experiences following PRS testing

Detailed demographic and clinical characteristics of the Swedish BRIGHT study arm will be presented in a separate clinical manuscript; in brief, the cohort of 792 women had a mean age of 37.4 years (SD 3.1; range 30–49) and a mean body mass index of 24.9 kg/m2 (SD 4.6), and was predominantly of European ancestry (94.2%; Asian 3.7%; other 2.1%). Most had been pregnant at least once (84.5%), 12.1% reported a previous breast biopsy or operation, and 1.0% had undergone previous genetic testing for breast cancer. Regarding family history of cancer, 10.9% reported a first-, second-, or third-degree relative with a known breast-cancer-predisposing mutation (44.9% reported none and 44.1% were uncertain), 28.8% reported a first- or second-degree relative with breast or metastatic prostate cancer, and 16.8% reported a relative diagnosed with breast cancer before age 51. Family history of cancer did not differ significantly across the PRS risk groups (χ² = 2.37, df = 2, *p* = 0.31), as reported in the companion clinical study paper. Among the 9-month (Survey 2) respondents who could be linked to their recruitment and PRS data (*n* = 281), self-reported understanding of the results and agreement that participation had been the right decision were uniformly high (at least 97% and 91% across subgroups, respectively) and did not differ significantly by PRS risk group (*p* = 0.16 and *p* = 0.16) or by family history (*p* = 0.31 and *p* = 0.51), including among the small subgroups with discordant family-history and PRS profiles (for example, family history of cancer with a low or average PRS, or an elevated PRS without family history). Education level and region of residence were not collected; references to participants’ educational background therefore derive solely from voluntary free-text comments.

Table 1 summarizes participants’ experiences after they have had their BC PRS-test results from Survey 1 and Survey 2. In Survey 1, conducted shortly after result disclosure (*n* = 400), participants reported generally positive emotional states: 85% felt calm and 78% felt comfortable, while few reported negative emotions, with only 15% experiencing worry, 13% feeling anxious, and 12% feeling nervous. This pattern generally persisted in the later Survey 2 (*n* = 289), where 84% continued to report feeling calm and 73% felt comfortable, with similarly low levels of negative emotions (18% worried, 16% anxious, 11% nervous).

Emotional responses appeared somewhat context-dependent over time. Based on the qualitative comments, participants often attributed current anxieties to external life stressors rather than the PRS result itself. However, initial qualitative feedback from Survey 1 highlighted that receiving a high-risk result could provoke significant immediate worry and a desire for prompt support among some participants.

Participants consistently valued the received PRS information, with 87% and 81% finding the results interesting in Survey 1 and 2, respectively and 82% finding them valuable in both surveys. However, comprehension ratings were more mixed: approximately 75% (297/397) agreed the explanations were understandable in Survey 1 and 82% (239/290) in Survey 2 (with 44% and 78%, respectively, agreeing fully). Although most participants rated the explanations understandable, qualitative data across both surveys revealed that a substantial subset struggled to interpret their results. A recurring theme in the free-text responses was the difficulty some participants experienced in understanding their results, with numerous comments describing the information as “svårt att tolka [hard to interpret],” “very academic,” using “svårt språk [difficult language],” and lacking clarity regarding personal risk implications or the specific meaning of statistical terms. Requests for simpler language (“förenklat provsvar”), less jargon, clearer visualizations, and explicit statements of risk category were frequent (appearing in 50% of all open-ended responses), even among highly educated participants. A second major theme emerging from the qualitative data from Survey 2 was uncertainty about the practical implications and required actions following the result. Participants frequently expressed a need for clear recommendations or an action plan tailored to their risk level.

Despite these comprehension challenges, the vast majority of participants viewed their participation positively. In Survey 2, 88% agreed that undergoing testing was the right decision and only 5% expressed any regret (Table 1).

**Table 1:** Participants’ experiences following PRS result disclosure

Domain	Survey 1 (*n* = 400)	Survey 2 (*n* = 289)	Key Qualitative Themes
**Emotional response**			
Felt calm	85%	84%	Generally positive emotional response maintained over time
Felt comfortable	78	73%	
Experienced worry	15%	18%	Most negative emotions were attributed to external stressors, not PRS results
Experienced anxiety	13%	16%	
Experienced nervousness	12%	11%	
**Information evaluation**			
Found information valuable	82%	84%	Information consistently valued despite comprehension issues
Found information interesting	87%	-	High interest maintained over time
Understood information well	~ 75%	~ 82%	Most agreed the explanations were understandable; free-text comments still noted difficulty with probabilistic/statistical terms
**Decision satisfaction**			
Made right decision	-	88%	Overwhelmingly positive about participation
Regret decision	-	5%	Minimal regret despite comprehension challenges
**Qualitative themes: major unmet needs**			
Clearer explanations	-	-	“Hard to interpret,” “very academic,” “difficult language”
Actionable guidance	-	-	“What am I expected to do with my result?”
Better follow-up support	-	-	Frustration with accessing promised consultations

Note: Percentages represent combined “agree” and “strongly agree” responses for positive statements, and “disagree” and “strongly disagree” for negative statements. Specific sample sizes varied by question due to non-response

Abbreviations: PRS = Polygenic Risk Score; - = Not Applicable

### Professional perspectives on PRS implementation

In the KAP survey, 5 of 6 respondents reported being familiar with the concept of PRS and 1 of 6 was not; familiarity with their region’s existing breast cancer screening guidelines was more varied (3 of 6 very familiar, 2 of 6 somewhat familiar, 1 of 6 not very familiar). Perceptions of PRS accuracy and reliability were cautious: 1 of 6 rated it moderately accurate and 5 of 6 were neutral or undecided, with none rating it highly accurate. All 6 respondents regarded integration of PRS as at least somewhat important (3 somewhat, 2 moderately, 1 very important), and all 6 considered that integration would improve early detection (5 somewhat improve, 1 significantly improve). Anticipated benefits included improved risk identification and potentially more efficient screening resource allocation.

The FGD highlighted the possibility of personalized (risk-stratified) screening pathways (Oncologist), in which the age at which screening begins and the frequency or modality of mammography are tailored to an individual’s PRS-based risk rather than determined by age alone. However, significant reservations were expressed about the clinical and ethical acceptability of reducing screening intensity based on low PRS scores (Breast Radiologist). FGD participants framed PRS primarily as a potential adjunct to existing clinical assessment, particularly for high-risk families initially (Medical Geneticist), rather than a standalone tool. Immediate concerns were raised in the FGD regarding the scientific evidence, specifically citing the AUC value of 0.6 and high chances of categorizing women into the wrong risk categories (Breast Radiologist). Significant barriers to implementation were consistently mentioned. The lack of established clinical guidelines emerged as a primary concern, closely linked to the perceived need for more robust evidence demonstrating clinical utility and cost-effectiveness (Biomedical Engineer, Breast Radiologist). Communicating complex, probabilistic risk information effectively to patients was viewed as a substantial challenge (Breast Radiologist), necessitating significant investment in provider education. System-level hurdles included anticipated costs and reimbursement issues, difficulties in integrating PRS into clinical workflows and electronic health records, and perceived inertia or lack of openness from regulatory or guidance bodies such as the Swedish Board of Health and Welfare (Socialstyrelsen) (Breast Radiologist). Ethical concerns regarding data privacy and potential patient anxiety were also mentioned (Biomedical Engineer).

Consequently, confidence in the healthcare system’s readiness to integrate PRS was limited: 1 of 6 respondents reported being moderately confident, 3 of 6 somewhat confident, and 2 of 6 not confident at all, with none very or extremely confident. Professionals universally stressed that large-scale clinical trials demonstrating utility and the subsequent development of clear, evidence-based guidelines were critical prerequisites for adoption. A potential timeline of approximately five years post-evidence availability was suggested for wider implementation (Oncologist, Breast Radiologist, Medical Geneticist) (Table 2).

**Table 2:** Professionals’ perspectives on PRS implementation based on the KAP survey and focus group discussion (*n* = 6)

Domain	KAP Survey Results	Key Concerns & Perspectives (FGD)
Current Familiarity	Familiar with the PRS concept: 5 of 6 yes, 1 of 6 no	Varied understanding of PRS definition; viewed as adjunct to existing tools rather than standalone solution
	67% not familiar	
Perceived Potential	Accuracy/reliability of PRS: 1 of 6 moderately accurate, 5 of 6 neutral/undecided, 0 of 6 high	Acknowledged utility for personalized screening pathways, but tempered by need for stronger evidence
	Integration considered at least somewhat important by 6 of 6 (1 very, 2 moderately, 3 somewhat); 6 of 6 expect it would improve early detection	
Major Implementation Barriers	**Unanimous concerns (6/6)**: • Lack of clinical guidelines • Need for utility evidence **Most cited (5/6)**: • Cost/reimbursement issues • Provider education needs • EHR integration challenges	**Technical concerns**: Concern about AUC value of 0.6 and high chances of categorizing women in wrong risk levels (Breast Radiologist) **System barriers**: the Swedish Board of Health and Welfare not open to PRS even for high-risk individuals (Breast Radiologist); rigid adherence to population vs. hybrid screening; difficulty communicating risk to laypeople **Evidence gaps**: Need clinical trials proving effectiveness and cost-effectiveness (Oncologist, Medical Engineer); Health Technology Assessment required
Implementation Readiness	Confidence in system readiness: 1 of 6 moderately confident	Readiness contingent on addressing evidence and guideline gaps
	3 of 6 somewhat confident, 2 of 6 not at all confident	
	0 of 6 very or extremely confident	
Prerequisites for Adoption	**Essential requirements (6/6)**: • Demonstrated clinical utility • Evidence-based guidelines	Timeline estimate: ~5 years after robust evidence becomes available Emphasis on need for large randomized trials and official gridlines before implementation
	**Important factors (5/6)**: • Cost-effectiveness data • Provider education programs	

KAP survey figures are counts out of six respondents; entries in the right-hand column are qualitative observations from the focus group discussion and are not survey counts. AUC = Area Under the Curve (predictive accuracy measure where 1.0 = perfect, 0.5 = random chance); EHR = Electronic Health Record; KAP = Knowledge, Attitudes, Practices; PRS = Polygenic Risk Score

### Synthesis across perspectives

The findings reveal a significant gap without clinical decision support between the technical availability of PRS information and its effective communication and clinical application within the study context. Professionals’ anticipatory concerns about communication difficulties were strongly mirrored in participants’ widespread reports of challenges to understand their results, despite valuing the information in general. This reveals a critical need for developing and implementing person-centered reporting methods and enhanced communication support. Furthermore, the professional emphasis on requiring clear clinical guidelines and defined care pathways aligns directly with participants’ expressed uncertainty about necessary actions post-result and their frustrations regarding access to follow-up. While PRS holds recognized potential, these communication and translation barriers currently limit its practical utility from both professional and patient viewpoints.

## Discussion

This mixed-methods study provides valuable insights into the perspectives surrounding the implementation of PRS for BC risk assessment within the Swedish healthcare context, highlighting both positive attitude and value from study participants and some barriers from the viewpoints of medical professionals, health system stakeholders, and participants who were not very comfortable with the interpretation of PRS results.

Our findings indicate that breast cancer PRS testing should be implemented only alongside clear, evidence-based clinical guidelines and standardized, integrated communication strategies. Given the complexity of the information involved and the current absence of such guidelines, PRS testing should be accompanied by integrated clinical decision support for both patients and healthcare professionals. A recently published guidance describes possible strategies to integrate PRS as a part of a person-centered healthcare system [30]. A key finding was the discrepancy regarding information comprehension. The remarkable consistency between professional concerns and participant experiences strengthens our findings. Professionals anticipated exactly the problems that participants encountered: difficulties understanding complex risk information, uncertainty about appropriate actions, and frustration with accessing follow-up support. Because professionals anticipated the same difficulties that participants subsequently reported (limited comprehension, uncertainty about next steps, and problems accessing follow-up), these barriers appear to reflect practical challenges experienced across stakeholder groups rather than theoretical concerns alone., This convergence was identified through side-by-side comparison of the themes arising in each group, not through statistical association.

The implementation of the BC PRS test, together with accompanying digital clinical decision support (software-based tools that translate the PRS result into actionable, person-centred guidance, such as automated screening recommendations by risk level, plain-language risk explanations, and information on modifiable risk factors) within a personalized BC screening service, has been described in the results of the Estonian arm of the BRIGHT study [23]. This approach has demonstrated high levels of satisfaction among women and a low need for post-test verbal counselling. The implemented clinical decision support includes a personalized recommendation on the appropriate age of initiation and frequency of mammographic screening according to individual risk, as well as information on modifiable non-genetic risk factors and guidance on breast awareness, including the possible early symptoms of breast cancer. These features directly target the comprehension and follow-up gaps observed in our Swedish cohort.

The limited familiarity with PRS among professionals in our study aligns with findings from other contexts, such as the UK, underscoring a widespread need for targeted clinician education before PRS can be used appropriately in routine practice [13]. To this end, some groups have begun to develop targeted educational materials and training resources to upskill clinicians in interpreting and communicating PRS [31–33]. Despite lack of experience, professionals saw significant potential utility. While professionals acknowledged the theoretical potential of PRS to refine risk stratification, their cautious approach was evident in multiple ways: immediate concerns about modest predictive values (AUC ~ 0.6) and patient misclassification risks, reluctance to consider screening de-intensification for lower-risk women, and low implementation readiness. This professional caution appears well-founded, given our participants’ feedback findings and echoes sentiments reported elsewhere [13], though it poses challenges to realising the full theoretical benefits of risk-stratified approaches.

The universally identified need among professionals for robust clinical utility evidence and authoritative clinical guidelines was strikingly validated by participants’ experiences. Our FGD participants’ specific concerns about communication difficulties and the lack of guidelines from bodies such as the National Board of Health and Welfare (Socialstyrelsen) proved prescient when examined against participants’ feedback. This alignment between professional predictions and participants’ experiences represents a notable finding that strengthens the case for addressing these barriers before wider implementation. Nevertheless, the expanding evidence on the feasibility and clinical utility of PRS-informed BC screening shows that large-scale implementation is achievable, leads to moderate adjustments in individual screening recommendations, and is expected to place only a minimal additional burden on the healthcare system [12]. Participants’ experiences in our study vividly illustrate the guidance gap at the individual level. Although approximately 85% of participants found the PRS information valuable and interesting, and most considered the explanations understandable (approximately 75% in Survey 1 and 82% in Survey 2), free-text responses revealed that a subset of participants still experienced difficulties interpreting the probabilistic risk information, echoing previously reported challenges in communicating complex risk information [15, 25]. Notably, comprehension difficulties were reported in both surveys and even by some highly educated individuals, including those with medical backgrounds and advanced university degrees. Comments such as “I have studied for quite a few years at university and work in healthcare” and “I have a medical university degree but still had to ponder a bit” suggest that current reporting formats were inadequate even for populations with above-average health literacy. This finding indicates that the communication challenge extends beyond typical health literacy concerns and represents a more fundamental issue with how probabilistic genetic risk information is presented and explained. The positive experience of our Estonian colleagues, who provided, in addition to written information, face-to-face pre- and post-test consultations upon request, demonstrates that supplementing written reports with optional consultation can further support understanding and satisfaction, a model worth considering alongside the email-based delivery used in our Swedish study [23].

While participants generally valued receiving the information and reported minimal lasting psychological harm, consistent with findings from other research settings [23, 34], the practical benefit was perceived as limited, in spite of the fact that women with medium and high PRS score were invited to book a consultation with further investigation. The fact that 88% of participants agreed that testing was the right decision, despite comprehension difficulties, suggests that the demand for personalised genetic risk information exists but requires more effective delivery mechanisms.

This disconnect between information provision and practical utility highlights that the clinical utility of PRS is not solely dependent on the test’s predictive power but also critically relies on the health system’s capacity to support patients in understanding and acting upon the information provided. The approximately five-year timeline suggested by professionals in the FGD (measured from the point at which robust clinical-utility evidence and guidelines become available, not from the present) appears realistic given the scope of changes required in communication strategies, clinical pathways, and provider education [35, 36].

The Swedish context offers potential advantages for BC PRS implementation, such as robust national registries and organized screening programs [37, 38], which could facilitate evaluation and delivery of risk-stratified approaches. However, our findings suggest that specific challenges within this system must be addressed before implementation can proceed effectively. The unanimous professional requirement for clinical guidelines and the participant needs for clearer actionable guidance point to the importance of developing Swedish-specific implementation frameworks. Additionally, the equity concerns raised globally regarding ancestry bias are pertinent to Sweden’s diverse population and would need careful consideration in any national implementation strategy, aligning with the strong equity focus typical of Nordic healthcare models [39, 40].

### Strengths and limitations

This study has several strengths, including its mixed-methods design integrating perspectives from clinicians, decision-makers, and a large cohort of participants (*n* = 400 in Survey 1, *n* = 289 in Survey 2) at two timepoints post-result disclosure, providing a nuanced picture within a specific national health system. The convergence of professional predictions with participants’ experiences strengthens the credibility to both sets of findings. However, limitations must be acknowledged. The sample size for the professional KAP survey and FGD was small (*n* = 6), potentially limiting the generalizability of these specific findings, although the themes resonated with broader literature. Participant recruitment occurred primarily through social media platforms, local media, and websites, which may have introduced selection bias toward more digitally engaged populations. Additionally, participants were recruited for a specific research study (BRIGHT) targeting healthy women aged 30–49, and their experiences might differ from those encountered in routine clinical practice. Response bias may also be present, as individuals with stronger opinions or particular negative experiences might be more likely to complete feedback surveys [41]. The analysis relied on descriptive statistics and thematic analysis, precluding causal inferences. Several further limitations should be noted. First, no baseline (pre-PRS) assessment of participants’ risk understanding or PRS knowledge was conducted; only post-result feedback surveys (at 4–6 weeks and 9 months) were administered, which precludes any pre- versus post-testing comparison of comprehension. Second, the two participant feedback surveys could not be linked at the individual level because the exports did not share a common identifier; consequently, the proportion of Survey 2 respondents who had also completed Survey 1 is based on self-report, and no paired, within-person change analysis was possible. Third, integration of the quantitative and qualitative strands occurred mainly at the interpretive level, and convergence was established through cross-source thematic comparison rather than statistical association. Fourth, the FGD was documented through structured observer notes rather than verbatim transcripts, without member checking or a formal audit trail. Finally, education level and region of residence were not formally ascertained. PRS results were delivered to participants by a single email; email opens were not tracked and no reminders were sent, which may have affected engagement with the results. The feedback surveys were also developed for this study and were not formally validated or piloted.

### Implications for research and practice

Future research in Sweden should prioritize developing and rigorously testing different person-centered communication strategies and reporting formats for PRS results, with particular attention to addressing comprehension barriers that persist even among highly educated populations. Larger-scale surveys are needed to assess PRS knowledge, attitudes, and educational needs among a broader range of Swedish healthcare professionals, particularly in primary care. Studies indicate that primary care providers represent an effective channel for communicating and interpreting PRS results for patients; however, this necessitates further educational initiatives [15]. A significant hurdle remains the perceived lack of interest or “clinical inertia” from higher regulatory authorities (e.g., Socialstyrelsen), who often prioritize long-term outcome data over the rapid adoption of emerging genomic tools [42]. To address this, future research must move beyond clinical validity and focus on presenting the “value proposition” of PRS through the lens of implementation science and health economics [43]. Demonstrating that PRS can lead to more efficient resource allocation—rather than simply increasing the volume of follow-up care—is essential for gaining administrative support within the Swedish healthcare model [44]. This requires evidence that PRS integration reduces the “opportunity cost” of screening low-risk individuals while capturing high-risk patients who might otherwise be missed by traditional age-based protocols [45].

Prospective studies evaluating different models for integrating PRS assessment and follow-up support within the existing Swedish healthcare infrastructure are warranted. Given the convergence of professional and patient perspectives on implementation barriers, intervention studies addressing communication, clinical pathways, and provider education simultaneously may be most effective. Critically, addressing the performance and validation of PRS in diverse ancestral groups within the Swedish population is essential for ensuring equitable implementation.

## Conclusion

Polygenic risk score testing for breast cancer represents a scientifically robust approach to improving risk stratification and holds clear promise for more personalized prevention and screening strategies. Participants reported positive experiences, valuing the information provided, expressing low decision regret, and showing strong interest in study participation, while healthcare professionals demonstrated high confidence in genetics-based risk screening models. To fully realize this potential within Swedish clinical practice, continued development is needed. Our study identified several specific gaps that should guide this work: a comprehension gap (although most participants rated the explanations understandable, a notable minority found their results difficult to interpret); the absence of clear clinical guidelines and defined care pathways; an unmet need for actionable, person-centred follow-up guidance; and limited clinician familiarity with, and readiness for, PRS. Addressing the comprehension gap will require refining approaches to risk communication, specifically developing plain-language, person-centred report formats with clearer visualizations and explicit risk-category statements, supplementing written reports with optional pre- and post-test consultations, and providing next-step guidance tailored to each risk level. Further implementation requires Sweden-specific clinical guidelines for the use of breast cancer PRS, which would form the basis for clinical decision support for both patients and healthcare professionals. Our findings underscore the opportunity for collaborative efforts among researchers, clinicians, policymakers, and patient representatives to create tailored implementation pathways, invest in education and communication tools, and integrate PRS in ways that support accessible, effective, and equitable healthcare.

## Supplementary Information

Below is the link to the electronic supplementary material.

Supplementary Material 1

## Data Availability

The data that support the findings of this study are available from the corresponding author, upon reasonable request.
