# Supplementary material for: A mixed-methods exploration of stakeholder experiences and perspectives on integrating polygenic breast cancer risk scores into Swedish clinical practice
Source: BMC Health Serv Res. 2026 Jul 11;26:948. doi: 10.1186/s12913-026-15116-7 (PMC13356793; doi:10.1186/s12913-026-15116-7)
Supplement: Supplementary file 1 — Supplementary Material 1 [file 12913_2026_15116_MOESM1_ESM.docx]

Appendix

*A Mixed-Methods Exploration of Stakeholder Experiences and Perspectives on Integrating Polygenic Breast Cancer Risk Scores into Swedish Clinical Practice*

## Appendix Contents

1. Figure A1: Sample AnteBC Polygenic Risk Score Report
2. Figure A2: Participant Result Letters by Risk Category
3. Appendix A: Participants’ Feedback Survey 1 (English Translation)
4. Appendix B: Participants’ Feedback Survey 2 (English Translation)
5. Appendix C: Professional Knowledge, Attitudes, and Practices (KAP) Survey
6. Appendix D: Focus Group Discussion (FGD) Guide

# Figure A1: Sample AnteBC Polygenic Risk Score Report

*Note: The report below is a de-identified example of the personalised AnteBC polygenic risk score report generated by OÜ Antegenes (Estonia) and delivered to participants via email. The original report is in Swedish. A summarised English description of its contents is provided below.*


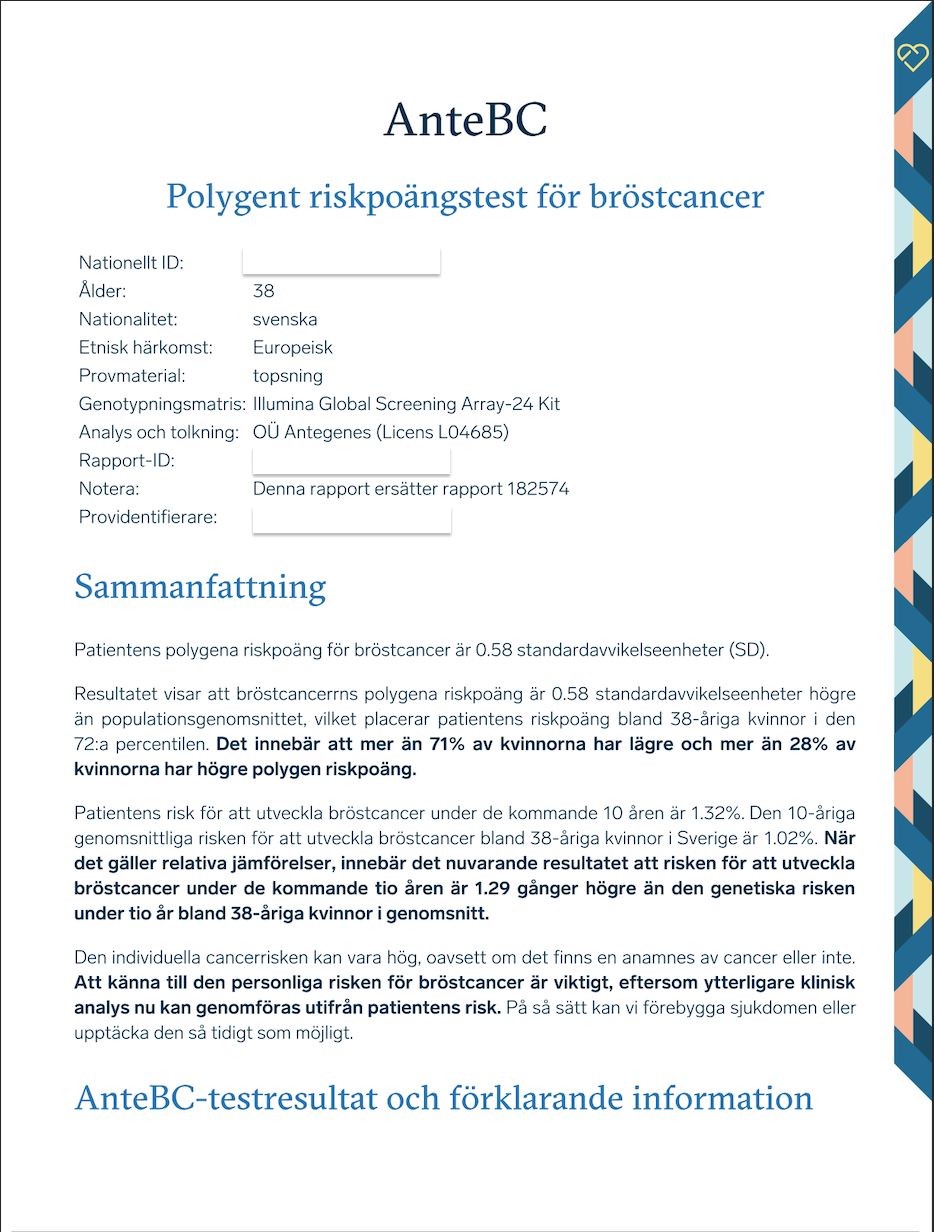


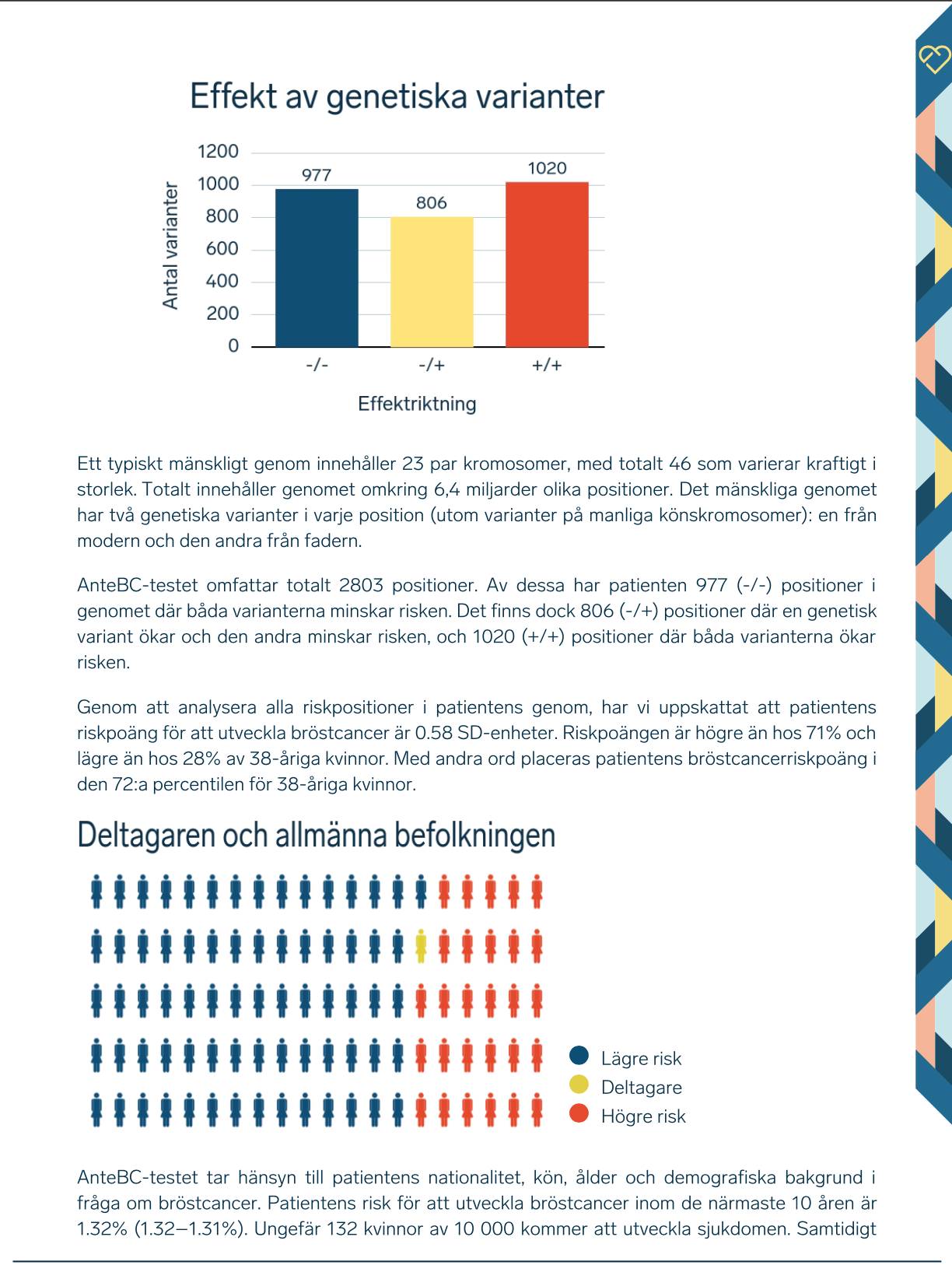


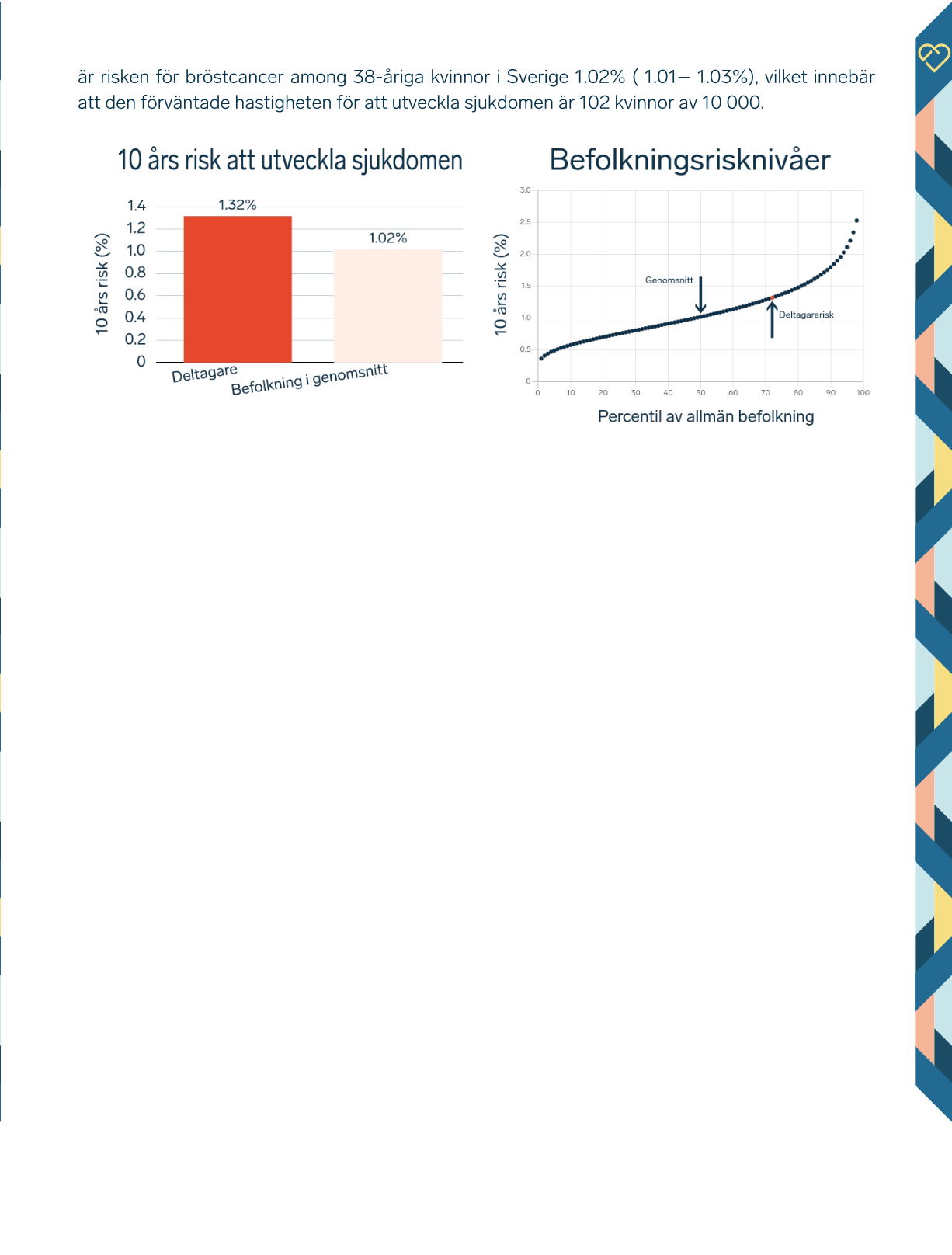


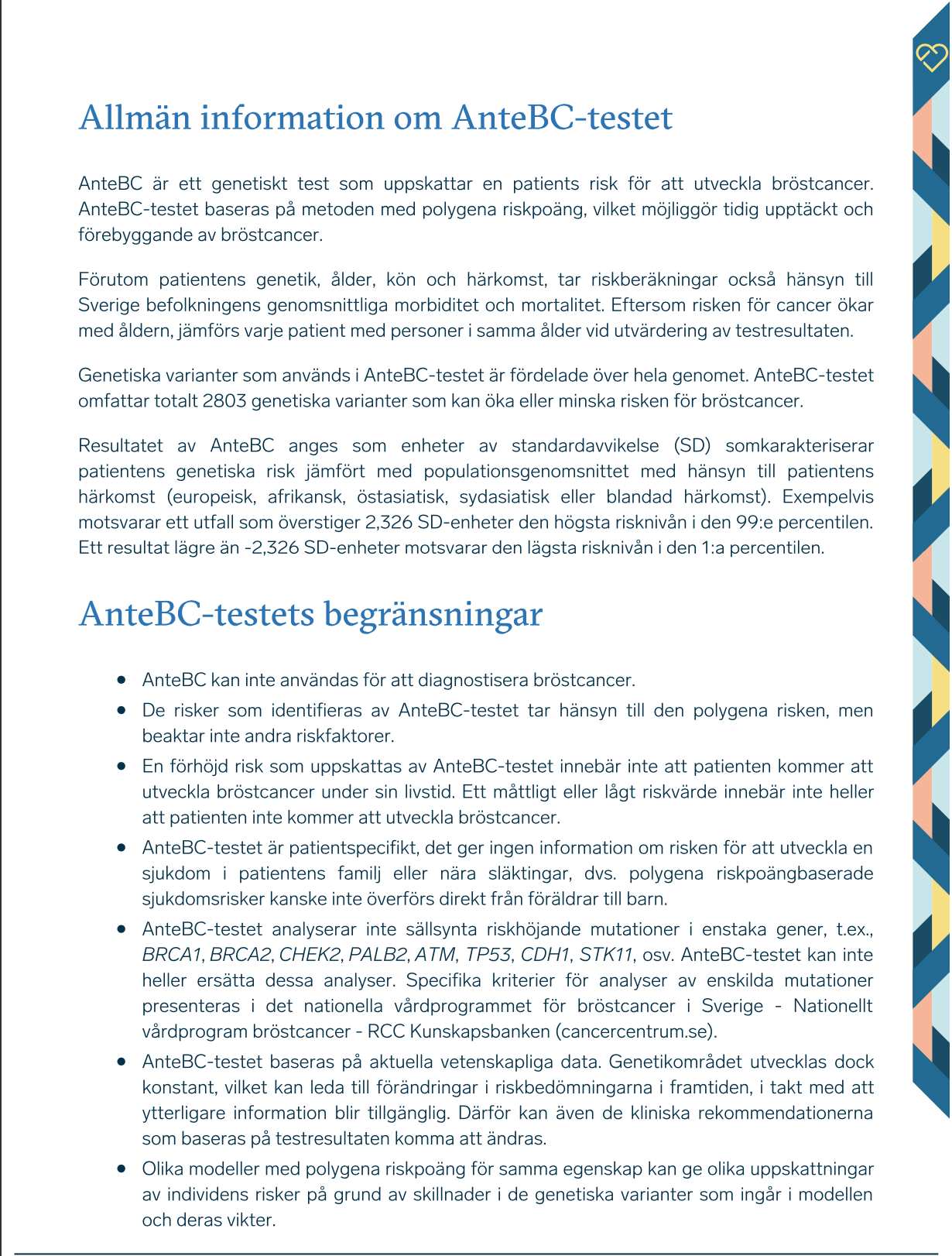


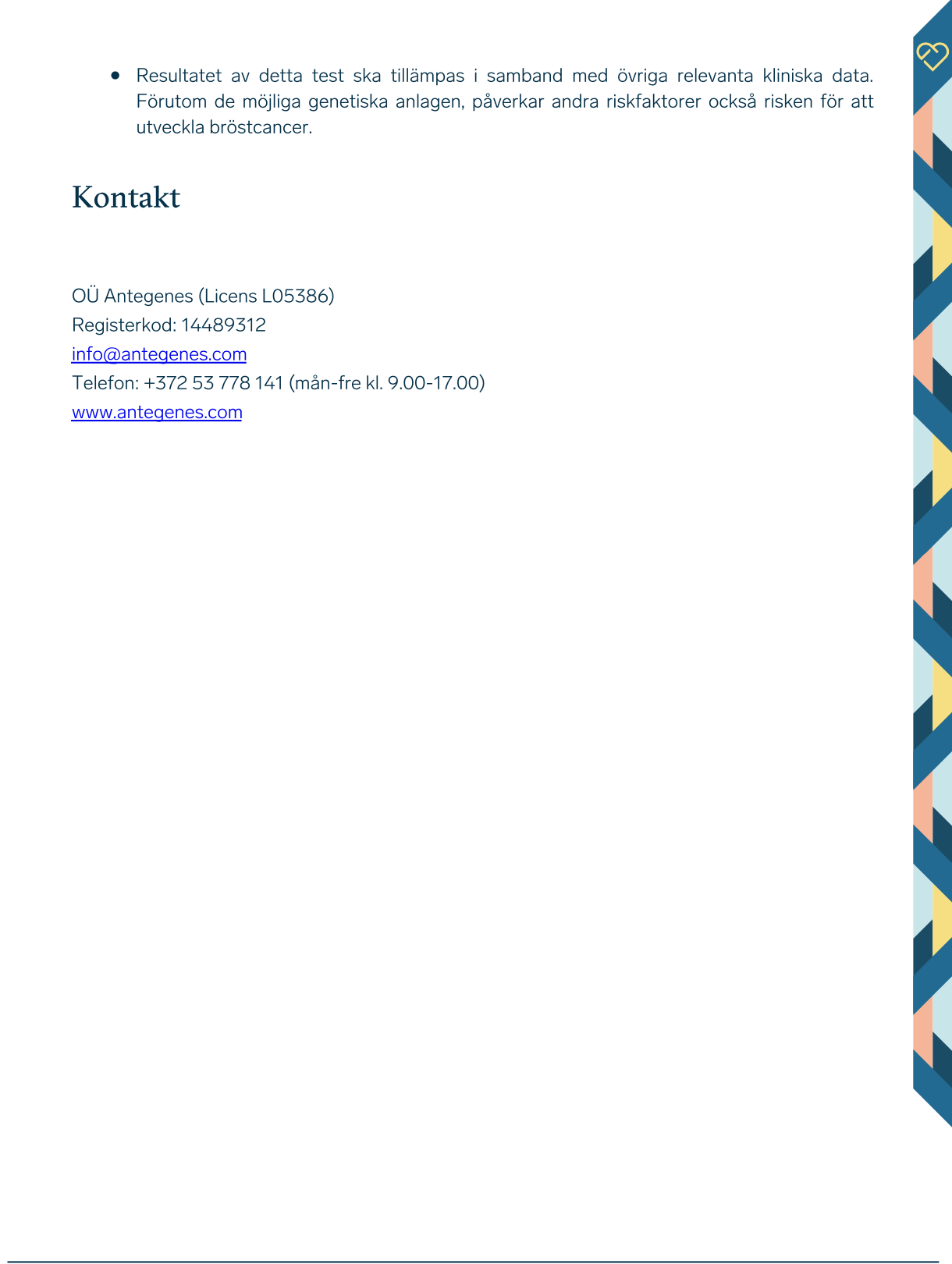


Figure A1. De-identified example AnteBC polygenic risk score report (5 pages), as delivered to participants. Personal identifiers have been removed.

## Report Contents (English Summary)

The AnteBC report (5 pages) contains the following sections:

**Page 1 – Summary:**

- Patient identifier, age, nationality, ethnic background, specimen type
- Genotyping platform: Illumina Global Screening Array-24 Kit
- Analysis and interpretation: OÜ Antegenes (License L04685)
- Polygenic risk score expressed in standard deviation (SD) units
- Percentile ranking among women of the same age
- Absolute 10-year breast cancer risk estimate (participant vs. population average)
- Relative risk comparison statement (e.g., "82% lower than the genetic risk among 39-year-old women on average")

**Page 2 – Effect of Genetic Variants:**

- Bar chart showing the number of genetic variants by effect direction: risk-decreasing (–/–), mixed (–/+), and risk-increasing (+/+)
- Explanation that the AnteBC test covers 2,803 genomic positions
- Population comparison pictogram showing the participant’s position relative to lower-risk (dark) and higher-risk (red) individuals

**Page 3 – Risk Visualisations:**

- Side-by-side comparison chart: participant’s 10-year risk vs. population average 10-year risk
- Population risk level curve showing the participant’s percentile position on a continuous risk distribution

**Page 4 – General Information about the AnteBC Test:**

- Description of the test methodology (polygenic risk score approach)
- Explanation of how age, sex, nationality, and demographic background are considered
- Information about the 2,803 genetic variants included in the score
- Interpretation of SD units and percentile rankings

**Page 4–5 – Limitations of the AnteBC Test:**

- The test cannot be used to diagnose breast cancer
- Polygenic risk does not account for other risk factors
- An elevated risk does not guarantee disease development; a low risk does not guarantee protection
- The test is patient-specific and does not directly predict risk for family members
- The test does not analyse rare high-penetrance mutations (e.g., BRCA1, BRCA2, CHEK2, PALB2, ATM, TP53, CDH1, STK11)
- Results are based on current scientific data and may be updated as knowledge advances
- Different PRS models may yield different risk estimates

**Page 5 – Contact Information:**

- OÜ Antegenes contact details (License L05386)

# Figure A2: Participant Result Letters by Risk Category

*Note: The following are English translations of the template letters sent to participants via email together with their AnteBC PRS report. Three versions were used, corresponding to the three risk categories. The original letters were in Swedish.*

## Risk Classification

Participants were classified into three risk categories based on their relative risk (RR) compared to the general population:

| **Risk Category** | **Relative Risk (RR)** |
| --- | --- |
| Low or average risk | RR < 1.5 |
| Slightly elevated risk | 1.5 ≤ RR < 2.7 |
| Elevated risk | RR > 2.7 |

## A2a. Letter for Low or Average Risk

Dear Participant,

You are receiving this message because you are participating in the BRIGHT study, in which you submitted a DNA sample (AnteBC test) to analyse your individual statistical risk of developing breast cancer.

Your result is now available and is attached to this email.

The result indicates your polygenic risk score (PRS), and in the table below you can see which risk group you belong to.

**For participants with low or average genetic risk according to the AnteBC test:**

Our recommendations for those with a low or average statistical risk are to examine your breasts regularly and to attend mammography screening when called (for those aged 40 and older).

**Important information about your AnteBC test result**

If your result shows a low or average statistical risk, this does not guarantee that you will not develop breast cancer during your lifetime. If your result shows a slightly elevated or elevated statistical risk, this does not automatically mean that you will develop breast cancer during your lifetime.

## A2b. Letter for Slightly Elevated Risk

Dear Participant,

You are receiving this message because you are participating in the BRIGHT study, in which you submitted a DNA sample (AnteBC test) to analyse your individual statistical risk of developing breast cancer.

Your result is now available and is attached to this email.

The result indicates your relative risk (row 9 under the heading "Summary"), and in the table below you can see which risk group you belong to.

**For participants with slightly elevated genetic risk according to the AnteBC test:**

We offer you the opportunity to book a consultation at the Breast Unit at Samariterhemmet. There you will meet a physician affiliated with the BRIGHT project to discuss your result and any potential preventive measures that may be relevant for you. The consultation is voluntary.

To book a consultation, call the Breast Unit and state that you are participating in the BRIGHT project.

If you do not wish to book a consultation, we recommend that you examine your breasts regularly and attend mammography screening when called (for those aged 40 and older).

## A2c. Letter for Elevated Risk

Dear Participant,

(Introductory text as in letter A2b above.)

**For participants with elevated genetic risk according to the AnteBC test:**

We offer you the opportunity to book a consultation at the Breast Unit at Samariterhemmet. There you will meet a physician affiliated with the BRIGHT project to discuss your result and any potential preventive measures that may be relevant for you. The consultation is voluntary.

To book a consultation, call the Breast Unit and state that you are participating in the BRIGHT project.

If you do not wish to book a consultation, we recommend that you examine your breasts regularly and attend mammography screening when called (for those aged 40 and older).

## Common Closing Text (All Letters)

About the BRIGHT Study

Breast cancer is the most common malignant tumour disease and cause of cancer death among women in Sweden. To detect breast cancer as early as possible and reduce mortality, mammographic screening is conducted in Sweden. Breast cancer detected early can be treated more effectively. Current screening targets women aged 40–74. Unfortunately, breast cancer also occurs in younger women, but it is not practical to screen everyone in the same way.

A modern approach is to conduct breast cancer screening based on individual risk assessments. Breast cancer is sometimes genetically determined, so genetic tests that assess risk can provide information about an individual woman’s risk of developing breast cancer. Knowing one’s individual statistical risk is important, as further clinical analysis can be carried out based on this risk, thereby improving opportunities for prevention or early detection.

The BRIGHT study includes healthy women aged 30–49 with no history of breast or ovarian cancer and no known hereditary predisposition for breast cancer.

The principal investigator in Sweden is Region Uppsala and Uppsala University. The study was approved by the Swedish Ethical Review Authority (Ref: 2022-03074-01).

Contact: bright@regionuppsala.se

# Appendix A: Participants’ Feedback Survey 1

*Note: This survey was originally administered in Swedish via REDCap. The English translation below was prepared by the authors. The original Swedish instrument is available as a supplementary file. This survey was administered 4–6 weeks after PRS result disclosure (n = 400).*

**BRIGHT Study Feedback Survey #1**

Dear Study Participant,

You are participating in the BRIGHT research project – Risk Assessment and Early Interventions for Breast Cancer, conducted by the University of Tartu, OÜ Antegenes, Region Uppsala, and Uppsala University. You have now received your result. By completing this questionnaire, you help us understand how you value the information you received. Please answer the following questions by selecting the response option that best reflects your opinion. We ask that you answer all questions.

1. Where did you learn about the BRIGHT project?

- Friend/acquaintance/family member
- Newspaper/magazine
- Social media
- Other (please specify)

1a. If other, where?

____________________________________________________________

2. Was the information about the BRIGHT project sufficient, clear, and comprehensible for you?

- Yes
- Fairly
- Not particularly
- No
- Hard to say

3. How comfortable did you feel using a digital tool to register for the study?

- Not satisfied at all
- The portal is useful, but generally I am not satisfied
- The portal is somewhat useful, but it also has several shortcomings
- I am mostly satisfied, but there are some shortcomings
- I am very satisfied with the portal

3a. If you were not satisfied, with what?

- Login
- Giving consent
- Understanding information about the project
- There were technical problems
- Other (please specify)

3b. If other, what?

____________________________________________________________

3c. Recommendations for improving the use of the digital tool:

____________________________________________________________

4. Were you able to answer the questions related to cancer occurrence in your family?

- Yes
- Fairly
- Not particularly
- No
- Hard to say

5. How are you feeling right now? Please read the following statements carefully and indicate the extent to which they apply to you. It is important that you answer based on how you feel right now. Choose the response option that best fits you.

|  | **Agree** | **Somewhat agree** | **Agree to some extent** | **Disagree** |
| --- | --- | --- | --- | --- |
| I am calm | O | O | O | O |
| I am nervous | O | O | O | O |
| I feel anxious | O | O | O | O |
| I feel comfortable | O | O | O | O |
| I feel satisfied | O | O | O | O |
| I am worried | O | O | O | O |

6. The explanations you were given about genetic risk in connection with this study and the recommendations were...

|  | **Agree** | **Somewhat agree** | **Agree to some extent** | **Disagree** | **Hard to say** |
| --- | --- | --- | --- | --- | --- |
| Comprehensible | O | O | O | O | O |
| Interesting | O | O | O | O | O |
| Informative | O | O | O | O | O |
| Valuable | O | O | O | O | O |
| Sufficient | O | O | O | O | O |

6b. If another choice on the previous question, what?

____________________________________________________________

7. I am glad I had the opportunity to participate in the BRIGHT study.

- Agree
- Somewhat agree
- Agree to some extent
- Disagree
- Hard to say

8. After receiving the breast cancer risk test results, did you feel you needed further consultation with a healthcare professional?

- Yes
- Partially
- Not particularly
- No
- Hard to say

9. What other information would you have liked to receive? (free text)

____________________________________________________________

10. If further medical measures are needed in the near future based on the test result, do you know where to turn?

- Yes
- Fairly
- More or less
- Not really
- No
- I do not need any medical measures in the near future

11. If you have additional comments, suggestions, or ideas, please leave them here:

____________________________________________________________

12. If you would like a response to your comments, please write your phone number or email address here:

____________________________________________________________

# Appendix B: Participants’ Feedback Survey 2

*Note: This survey was originally administered in Swedish via REDCap. The English translation below was prepared by the authors. The original Swedish instrument is available as a supplementary file. This survey was administered approximately 9 months after PRS result disclosure (n = 289).*

**BRIGHT Study Feedback Survey #2**

Dear Study Participant,

You are participating in the BRIGHT research project – Risk Assessment and Early Interventions for Breast Cancer, conducted by the University of Tartu, OÜ Antegenes, Region Uppsala, and Uppsala University. We have sent you the results of the study. By completing this questionnaire, you help us understand how you value the information you received. Your opinion is very important to us. Please answer the following questions by selecting the response option that best reflects your opinion. We ask that you respond to all statements.

1. How are you feeling right now? Please read the following statements carefully and determine the extent to which they apply to you. It is important that you choose the answer based on how you feel right now. Choose the option that best fits you.

|  | **Agree** | **Somewhat agree** | **Agree to some extent** | **Disagree** |
| --- | --- | --- | --- | --- |
| I am calm | O | O | O | O |
| I am nervous | O | O | O | O |
| I feel anxious | O | O | O | O |
| I feel comfortable | O | O | O | O |
| I feel satisfied | O | O | O | O |
| I am worried | O | O | O | O |

2. For each statement, select the response that best fits you:

|  | **Agree** | **Partially agree** | **Agree to some extent** | **Disagree** | **Hard to say** |
| --- | --- | --- | --- | --- | --- |
| Knowing my genetic risks is something I can handle | O | O | O | O | O |
| The information I received changed my life in some way | O | O | O | O | O |
| I now have better access to healthcare | O | O | O | O | O |

3. How do you assess the following statements when you think back on your decision to receive the results of the genetic risk assessment? Please choose the response option that best fits.

|  | **Agree** | **Partially agree** | **Agree to some extent** | **Disagree** | **Hard to say** |
| --- | --- | --- | --- | --- | --- |
| It was the right decision | O | O | O | O | O |
| I regret my decision | O | O | O | O | O |
| I would make the same decision if I had to decide again | O | O | O | O | O |
| The decision caused me a lot of harm | O | O | O | O | O |
| I made a wise decision | O | O | O | O | O |

4. How do you assess your willingness to continue following the recommended follow-up plan?

|  | **Agree** | **Partially agree** | **Agree to some extent** | **Disagree** | **Hard to say** |
| --- | --- | --- | --- | --- | --- |
| I intend to follow the recommended follow-up plan | O | O | O | O | O |

5a. The recommendations and explanations you received in connection with the study were:

|  | **Agree** | **Partially agree** | **Agree to some extent** | **Disagree** | **Hard to say** |
| --- | --- | --- | --- | --- | --- |
| Comprehensible | O | O | O | O | O |
| Interesting | O | O | O | O | O |
| Informative | O | O | O | O | O |
| Valuable | O | O | O | O | O |
| Sufficient | O | O | O | O | O |

5b. If other recommendations, which?

____________________________________________________________

6. If you had a consultation after the test, how would you rate its usefulness?

- It was not useful at all
- It was somewhat useful
- It was generally useful
- It was very useful
- It was extremely useful

7. Have you shared the information and experience you gained during your participation in personalised breast cancer screening? With whom?

- Family member
- Friend
- Doctor
- On social media or in the press
- Other (please specify)
- I have not shared

7a. If shared elsewhere, where?

____________________________________________________________

8. Where have you looked for additional information? (Select all that apply)

- Asked a doctor
- Internet
- Other
- Have not searched

8a. If other, please specify:

____________________________________________________________

9. Do you have any recommendations or suggestions for what the study team could have done better?

____________________________________________________________

10. If you would like a response to your comments, please write your phone number or email address here:

____________________________________________________________

11. Did you respond to the first feedback survey?

- Yes
- No

# Appendix C: Professional Knowledge, Attitudes, and Practices (KAP) Survey

*Note: This survey was administered in English via REDCap to healthcare professionals and decision-makers (n = 6). The instrument is reproduced below as administered.*

**Polygenic Risk Scores in Breast Cancer Screening**

Dear Participant,

We appreciate your participation as a stakeholder representing various backgrounds, including health professionals, policymakers, decision-makers, and patient advocates.

This survey aims to gather insights on your knowledge, attitudes, and practices regarding PRS. Your responses will help us understand the current landscape and inform future efforts to integrate PRS into breast cancer screening guidelines.

Please answer the questions honestly and accurately. Your responses will be treated confidentially and used only for research purposes.

Your participation is voluntary, and you may withdraw at any time. By continuing, you give your consent to participate.

1. What is your professional background?

- Health professional (doctor, nurse, genetic counselor, etc.)
- Researcher/Scientist
- Policymaker/Decision-maker
- Patient advocate/Non-profit organization representative
- Other (please specify)

2. In what capacity are you participating in this survey?

- Healthcare provider/Practitioner
- Researcher/Scientist
- Policymaker/Decision-maker
- Patient advocate/Non-profit organization representative
- Other (please specify)

3. Are you familiar with the concept of Polygenic Risk Scores (PRS) in the context of breast cancer screening?

- Yes
- No

4. How would you define Polygenic Risk Scores (PRS) in the context of breast cancer screening? Please provide a brief description.

____________________________________________________________

5. What sources of information have you relied upon to learn about Polygenic Risk Scores (PRS) in the context of breast cancer screening? (Select all that apply)

- Scientific journals and publications
- Professional conferences and seminars
- Online resources (websites, forums, etc.)
- Healthcare professionals (doctors, genetic counselors, etc.)
- Colleagues or peers in the field
- Other (please specify)

6. What are your perceptions of the accuracy and reliability of Polygenic Risk Scores (PRS) as a screening method for breast cancer?

- Highly accurate and reliable
- Moderately accurate and reliable
- Neutral/Undecided
- Not very accurate and reliable
- Not at all accurate and reliable

7. To what extent do you believe the integration of Polygenic Risk Scores (PRS) into breast cancer screening guidelines would improve early detection and prevention?

- Significantly improve
- Somewhat improve
- Neutral/Undecided
- Not significantly improve
- Not at all improve

8. What are the potential benefits and limitations of incorporating Polygenic Risk Scores (PRS) into breast cancer screening guidelines? Please explain.

____________________________________________________________

9. How important do you consider the integration of Polygenic Risk Scores (PRS) into breast cancer screening guidelines?

- Not important at all
- Somewhat important
- Moderately important
- Very important
- Extremely important

10. What factors should be considered when determining the inclusion of Polygenic Risk Scores (PRS) in breast cancer screening guidelines? (Select all that apply)

- Accuracy and validity of PRS
- Availability and affordability of PRS testing
- Equity considerations in access to PRS testing
- Impact on screening resources and healthcare budget
- Potential benefits to high-risk populations
- Other (please specify)

11. What concerns or challenges do you foresee in implementing Polygenic Risk Scores (PRS) in breast cancer screening guidelines? (Select all that apply)

- Privacy and data security concerns
- Ethical considerations
- Limited knowledge and understanding among healthcare professionals
- Potential overuse or misuse of PRS results
- Inequitable access to PRS testing and resources
- Other (please specify)

12. How confident are you in the readiness of healthcare systems to incorporate Polygenic Risk Scores (PRS) into breast cancer screening guidelines?

- Not confident at all
- Somewhat confident
- Moderately confident
- Very confident
- Extremely confident

13. How likely are you to support the addition of Polygenic Risk Scores (PRS) to breast cancer screening guidelines in the future?

- Very likely to support
- Somewhat likely to support
- Neutral/Undecided
- Somewhat unlikely to support
- Very unlikely to support

14. Have you personally communicated with patients or the public about Polygenic Risk Scores (PRS) and their potential role in breast cancer screening?

- Yes: frequently
- Yes: occasionally
- No: but I have provided information when requested
- No: I have not had any such communication
- Not applicable to my role/background

15. How familiar are you with your region or organization’s existing breast cancer screening guidelines?

- Very familiar
- Somewhat familiar
- Neutral/Undecided
- Not very familiar
- Not at all familiar

16. In your opinion, what steps should be taken to ensure the successful integration of Polygenic Risk Scores (PRS) into breast cancer screening guidelines?

- Enhancing education and awareness among healthcare professionals
- Establishing clear guidelines and protocols for PRS utilization
- Conducting further research to validate PRS accuracy and effectiveness
- Ensuring equitable access to PRS testing and resources
- Engaging stakeholders in policy discussions and decision-making
- Other (please specify)

17. If Polygenic Risk Scores (PRS) were added to breast cancer screening guidelines, how likely would you be to incorporate PRS testing into your clinical practice or organization’s screening protocols?

- Very likely
- Somewhat likely
- Neutral/Undecided
- Somewhat unlikely
- Very unlikely

18. How important do you believe it is to include guidelines for genetic counseling alongside integrating Polygenic Risk Scores (PRS) into breast cancer screening guidelines?

- Not important at all
- Somewhat important
- Moderately important
- Very important
- Extremely important

19. What are the potential barriers or challenges that healthcare professionals may face when implementing Polygenic Risk Scores (PRS) in breast cancer screening guidelines?

____________________________________________________________

# Appendix D: Focus Group Discussion (FGD) Guide

*Note: The focus group discussion was conducted on June 13, 2023, lasting approximately 1.5 hours, with six professionals immediately following their completion of the KAP survey. The discussion was facilitated by one moderator, with three designated observers/note-takers. The guide was developed and administered in English.*

## Introduction

Welcome and introductions: Begin the session by welcoming the participants and introducing yourself and your team. Provide a brief overview of the purpose of the focus group, which is to gather insights and perspectives on introducing Polygenic Risk Scores (PRS) as part of breast cancer screening guidelines/protocols.

Explanation of the focus group process: The session will involve questions and open discussions. Assure participants that all responses will be confidential and encourage open and honest dialogue.

Ground rules: Emphasize the importance of respectful communication, active listening, and allowing everyone to express their opinions. Encourage participants to build upon one another’s responses and avoid dominating the discussion.

## Warm-up Question

“To get us started, could you briefly share your role or connection to breast cancer screening?”

## Main Questions

*Note: These questions can be rephrased or modified based on the flow of the discussion and participants’ responses.*

**1.** If you were leading this project, what steps would you take to ensure its success?

**2.** Which stakeholders do you consider crucial for successfully integrating PRS into breast cancer screening guidelines?

**3.** How can we gain support from healthcare professionals for this project? What potential concerns do you anticipate regarding the scientific aspects of PRS? What type of evidence would be necessary to address these concerns?

**4.** What other prerequisites do you think are necessary to incorporate PRS into the existing guidelines for breast cancer screening?

**5.** How would you envision the ideal implementation of PRS in breast cancer screening? What key components do you believe are essential in this perfect scenario?

**6.** Regarding the implementation process, what would be a reasonable timeline for adopting PRS in breast cancer screening?

**7.** What external factors or limitations do you anticipate that could affect the integration of PRS into the guidelines? What implementation challenges do you foresee concerning PRS?

**8.** Question for the representative of the advocacy group: What factors do you believe are most significant to patients concerning PRS and breast cancer screening?

**9.** Are there any ethical and/or legal considerations associated with using PRS in breast cancer screening that you see as noteworthy?

## Wrap-up

Summary: Summarize the main points discussed during the focus group, highlighting key insights and perspectives shared by the participants.

Additional comments: Allow participants to provide additional comments or insights regarding PRS and breast cancer screening.

Appreciation: Thank the participants for their valuable input and time in contributing to the discussion.

Next steps: Briefly mention the next steps in the research process, such as analysing the data collected and using the insights gained to inform future decisions.
